# Treatment of eruptive keratoacanthoma in the setting of immunosuppression and atopic dermatitis

**DOI:** 10.1016/j.jdcr.2026.02.025

**Published:** 2026-02-19

**Authors:** Adelle Pacyna, Rachel Cahn, Erica Ghareeb, Joanna Kolodney, Vlad Codrea

**Affiliations:** a School of Medicine, West Virginia University, Morgantown, West Virginia; b Department of Dermatology, West Virginia University, Morgantown, West Virginia; c Department of Medical Oncology, West Virginia University, Morgantown, West Virginia

**Keywords:** acitretin, dupilumab, eruptive keratoacanthoma, immunosuppression, koebnerization, squamous cell carcinoma

## Introduction

Eruptive keratoacanthoma (KA), a histological subtype of cutaneous squamous cell carcinoma, presents as multiple scattered crateriform lesions composed of well-differentiated keratinocytes with a central keratotic plug, an eosinophilic infiltrate, and intraepidermal neutrophilic abscesses.^1^^,^^2^ The etiology of eruptive KA is multifactorial, with predisposing factors including trauma, radiation exposure, immunosuppression, certain medications, human papillomavirus infection, heritable mutations, and autoimmune conditions such as vitligo.^2^^,^^3^

## Case report

**Fig 1:**
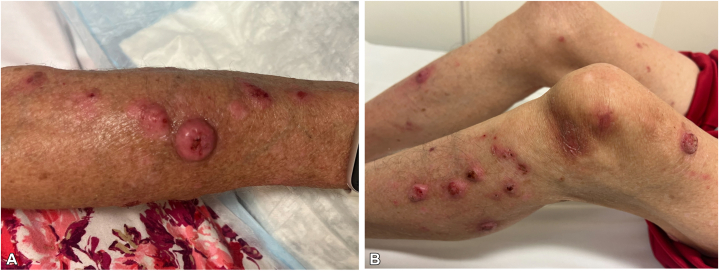
Clinical photos from the initial presentation. The patient exhibited diffusely scattered erythematous, tender, crateriform nodules involving the bilateral **(A)** upper and **(B)** lower extremities.

**Fig 2:**
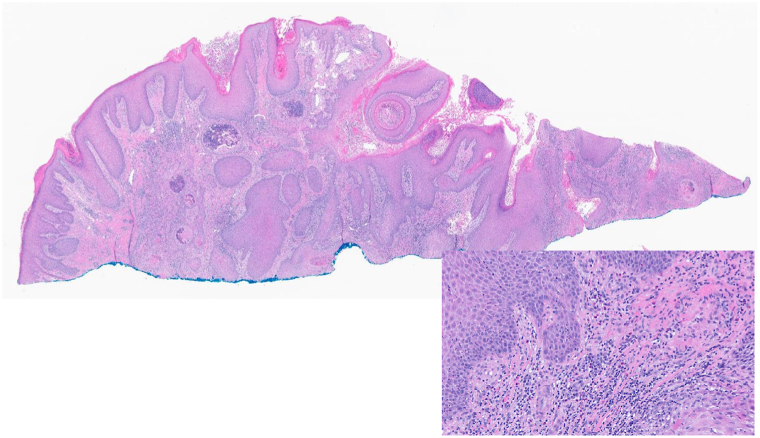
KA-type SCC. Histology of the lesion from the left arm revealed an endophytic proliferation of well-differentiated atypical keratinocytes with ground glass cytoplasm, invasion into the dermis, and an eosinophilic infiltrate (H&E 1× and inset 20×). *KA*, Keratoacanthoma; *SCC*, squamous cell carcinoma.

**Fig 3:**
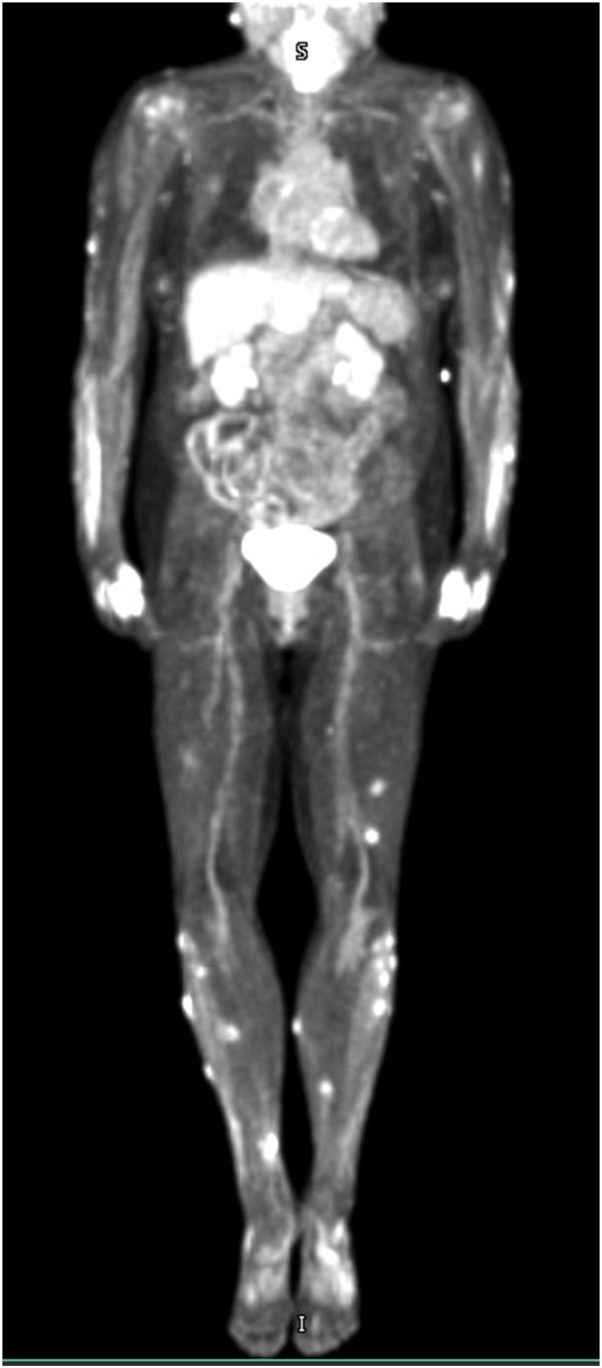
PET-CT body. Imaging showed diffuse hypermetabolic skin lesions concerning for malignancy, but no focal hypermetabolic activity in the brain, chest, abdomen, or pelvis to suggest internal metastasis. *PET-CT,* Positron emission tomography-computed tomography.

We describe the case of a 78-year-old female with a history of atopic dermatitis, diabetes mellitus, and long-term immunosuppression with tacrolimus following a heart transplant 25 years ago, who presented with painful nodules on the arms and legs (Fig 1). Biopsy showed KA-type, well-differentiated invasive squamous cell carcinoma (Fig 2). Positron emission tomography-computed tomography showed metabolically active lesions restricted to the skin. While cutaneous KAs can produce diffuse uptake on positron emission tomography-computed tomography, there was no systemic involvement (Fig 3).

**Fig 4:**
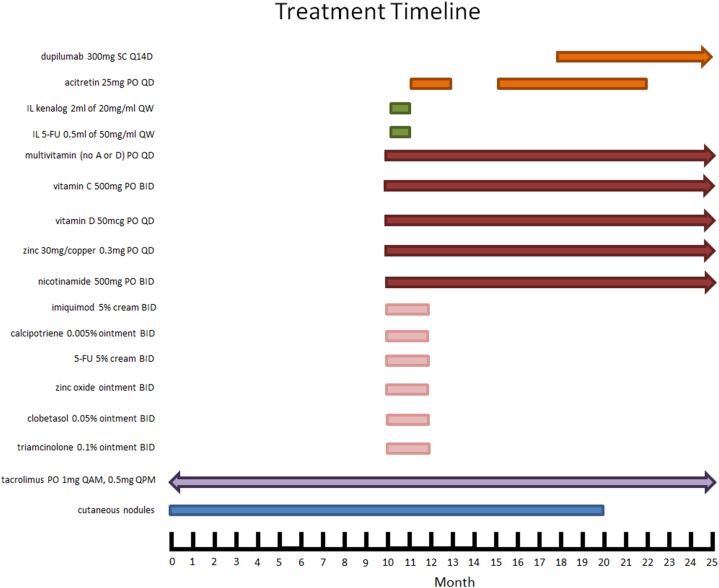
Timeline of important events in the management of the eruptive KA. *KA*, Keratoacanthoma.

Due to the substantial number and size of the nodules, surgical excision was not feasible. Instead, topical, intralesional, and systemic treatments were initiated as part of a tiered treatment plan (Fig 4). The tiers were designed to have increasing aggressiveness and risk of adverse effects and included modalities that had been previously shown to be effective in the treatment of KA. Tier 1 consisted of 5-fluorouracil cream mixed with calcipotriene, imiquimod, topical corticosteroid ointments (triamcinolone, clobetasol), and zinc oxide ointment. The patient was also nutritionally optimized with the following oral supplements: nicotinamide 500 mg twice daily, zinc 30 mg/copper 0.3 mg daily, vitamin D 50 mcg daily, vitamin C 500 mg twice daily, and a multivitamin daily (without vitamins A or D).

The patient reported difficulty sleeping due to burning sensations at the sites of chemotherapy cream application. Given the lack of efficacy and tolerability to Tier 1 treatments, Tier 2 therapy was introduced, consisting of intralesional injections. Selected lesions were injected weekly with triamcinolone or 5-fluorouracil. Methotrexate injections were considered but deferred due to interaction with trimethoprim-sulfamethoxazole, which the patient was taking for *Pneumocystis jirovecii* prophylaxis. The patient had difficulty tolerating the injections because of nausea and vomiting several hours afterwards, so Tier 3 treatment with oral acitretin 25 mg daily was initiated and maintained at this dose and frequency for the duration of its administration.

**Fig 5:**
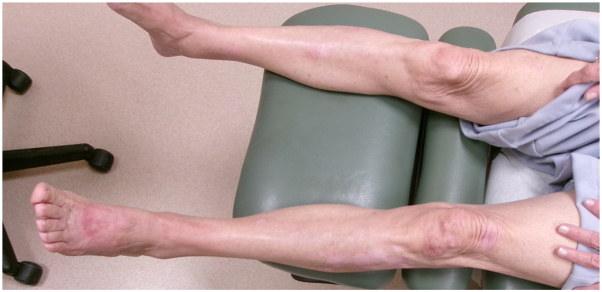
Post-treatment. Following combination treatment with acitretin and dupilumab, the patient achieved resolution of skin lesions without recurrence.

Throughout the treatment course, the patient endorsed intractable pruritus secondary to pre-existing atopic dermatitis and the topical therapies used during the Tier 1 treatment. The itching primarily involved her arms and legs, which coincided with the KA locations. Acitretin was held on multiple occasions and was ultimately discontinued before dose escalation could be attempted, because of xerosis and worsening atopic dermatitis. The xerosis caused severe cracking of the skin on the feet, which resulted in a left foot wound that became infected with *Pseudomonas aeruginosa* and *Staphylococcus aureus* and that required intravenous antibiotics to be administered during a multiday hospitalization. Because the pruritus and xerosis showed no improvement with topical steroid ointments, pramoxine cream, or dilute bleach baths, systemic dupilumab was initiated. The size and number of KA nodules gradually decreased starting with the acitretin administration and continued to decrease after dupilumab was added and after acitretin was stopped. Following initiation of dupilumab, the patient reported resolution of her pruritus and clearance of atopic dermatitis and KA nodules. She continues to take dupilumab and has not had recurrence of KA lesions 18 months later (Fig 5).

## Discussion

With the patient’s complex medical history, the etiology of eruptive KA was likely multifactorial. In an analysis of malignancy risk associated with immunosuppressive medications after solid organ transplantation, skin cancer was the most common malignancy. Additionally, heart and lung transplant recipients exhibited the highest overall rates of malignancy.^4^

Acitretin, a systemic retinoid, has been shown to be effective in the treatment of KA but can cause significant, dose-dependent, and often irreversible side effects.^5^ While the KA lesions improved on acitretin and may have continued to improve with extension of treatment duration or an increased dose, acitretin needed to be stopped due to severe side effects before the lesions resolved. Furthermore, alcohol is contraindicated during acitretin therapy as it facilitates the conversion of acitretin to etretinate, which is a metabolite with a lower therapeutic index and longer half-life. Although this 78-year-old patient is not at risk for retinoid-induced teratogenicity, the prolonged exposure to etretinate can increase the risk of other side effects such as cramps, dry mucous membranes, and skeletal abnormalities.

Dupilumab, a monoclonal antibody targeting the interleukin-4 receptor alpha subunit, blocks interleukin-4 and interleukin-13 signaling, suppressing Th2-mediated inflammation characteristic of atopic dermatitis, asthma, eosinophilic esophagitis, chronic rhinosinusitis with nasal polyposis, and prurigo nodularis. Its onset of action is relatively quick, starting to suppress pruritus over the entire body within 2 weeks.^6^ Because this patient experienced intractable pruritus from pre-existing atopic dermatitis, and eruptive KA can in part be precipitated by trauma, the suppression of itching proved to be a valuable adjunct treatment. Previous reports have described eruptive KA development in the setting of prurigo nodularis, suggesting that hypersensitivity and chronic pruritus may play a role in pathogenesis.^7^ While prurigo nodules may clinically appear similar to KA, they lack the central keratotic plug and the voluminous proliferation of glassy keratinocytes. Scratching and the subsequent wound healing response is a documented trigger for keratinocytic proliferation.^8^ Although previous reports have associated dupilumab with the development of eruptive KA,^9^^,^^10^ in this patient, dupilumab was used to suppress what was considered to be an underlying driver of the condition, namely scratching and subsequent koebnerization. This approach was taken due to the striking observation that all the patient’s lesions appeared on body areas that were easily accessible to scratching, which the patient was frequently observed to engage in during office visits.

The seemingly contradictory effects of dupilumab on eruptive KA between the previous case reports and this patient might point to a mechanistic difference in tumerigenesis. It is possible that dupilumab facilitates the development of eruptive KA in some patients while mitigating the risk factors in others. The patient described here represents only 1 case report of dupilumab aiding in the clearance of eruptive KA, so causality cannot be definitively established, and further research is needed.

### Declaration of generative AI and AI-assisted technologies in the writing process

AI was not used.

## Conflicts of interest

None disclosed.
